# Schmorl node induced multiple radiculopathy: A rare case report

**DOI:** 10.1097/MD.0000000000022792

**Published:** 2020-10-23

**Authors:** Yongjie Chen, Guojun Wei, Zongguang Li, Naichun Yu, Fengqing Gong, Guangrong Ji

**Affiliations:** Department of Orthopedic Surgery, Xiang’an Hospital of Xiamen University, Xiamen City, Fujian Province, China.

**Keywords:** disc herniation, radiculopathy, Schmorl nodes

## Abstract

**Rationale:**

We report a case of Schmorl node induced multiple radiculopathy.

**Patient concerns:**

A 70-year-old female patient complained of lower back pain in the left leg accompanied by numbness and weakness.

**Diagnosis:**

Radiographs showed obvious osteoporosis in the lumbar vertebrae. Computed tomography demonstrated a hole in the upper posterior half of the L2 vertebral body. Magnetic resonance imaging of the lumbar spine revealed a herniated disc involving a protrusion at the posterior wall of the L2 vertebral body, which was present in the left lateral and dorsal epidural spaces. There was significant lumbar stenosis at the L2 vertebral body secondary to dural sac compression due to the mass.

**Intervention:**

Left-sided hemilaminectomy was performed at L2 with screw fixation at L1–3. Intraoperatively, the severely ruptured disc compression in the dural sac and nerve root was removed.

**Outcomes:**

The patient's leg pain was immediately resolved, and her back pain was reduced. The patient recovered normal motor function at 20 days after surgery.

**Lessons:**

A Schmorl node can progress and break through the lumbar vertebral body, resulting in nerve compression. A large proximal herniated mass can cause distal multiple radiculopathy. Therefore, this special case of Schmorl node with multiple radiculopathy should be treated by removing the proximal herniated nucleus pulposus from the vertebral body.

## 1 Introduction

The most common source of lower back pain and sciatica is lumbar disc herniation, which is characterized by the displacement of the nucleus, cartilage, and fragmented apophyseal bone beyond the intervertebral disc space.^[1,2]^ The categories of herniation include protrusion, extrusion, sequestration, and migration. These are defined according to the difference in the distance between the edges of the disc material protruding outside the disc space and the distance between the edges of the base of the disc material extending outside the disc space.^[2]^ Another type of herniation, Schmorl node, is referred to as intervertebral herniation, which is defined as the herniation of the nucleus pulposus through the cartilaginous and bony endplate into the body of an adjacent vertebra.^[3]^ The great majority of patients with symptomatic Schmorl nodes present with only axial back pain related to inflammatory changes and cell infiltration induced by the presence of intraspongious disc components which contact with the vertebral bone marrow.^[4]^ Although some studies have described radiculopathy caused by Schmor node,^[5,6]^ multiple radiculopathy resulting from Schmorl node is a very rare condition. Here, we report a case of transcerebral intraspinal Schmorl node causing back pain and radiculopathy.

## 2 Case report

A 70-year-old female patient complained of lower back pain irradiating along the left leg accompanied by numbness and weakness that had lasted 3 weeks. The symptoms worsened over 10 days. Physical examination was performed. It was difficult for the patient to walk, and movement of the lumbar spine was mildly limited. There was obvious L4/L5/S1 and left paraspinal tenderness. There was decreased sensation on the anterior and posterior skin of the left leg. Manual muscle testing and a bilateral assessment of the deep tendon reflexes revealed the following results: quadriceps (III/V), tibialis anterior (V/V), extensor halluces longus (IV/V), flexor halluces longus (left III/V, right IV/V), patellar tendon reflex (normal/normal), and Achilles tendon reflex (normal/normal). The Lasegue sign test result was negative.

**Figure 1:**
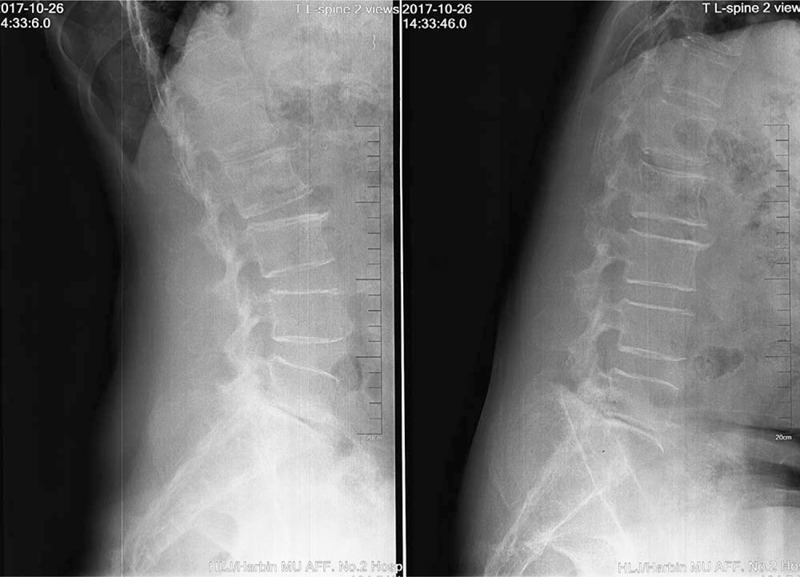
Radiographs showing obvious osteoporosis in the vertebrae and osteosclerosis in the endplates of L2, L3, and L4. Osteosclerosis was also found in the upper posterior half of the L2 vertebral body.

**Figure 2:**
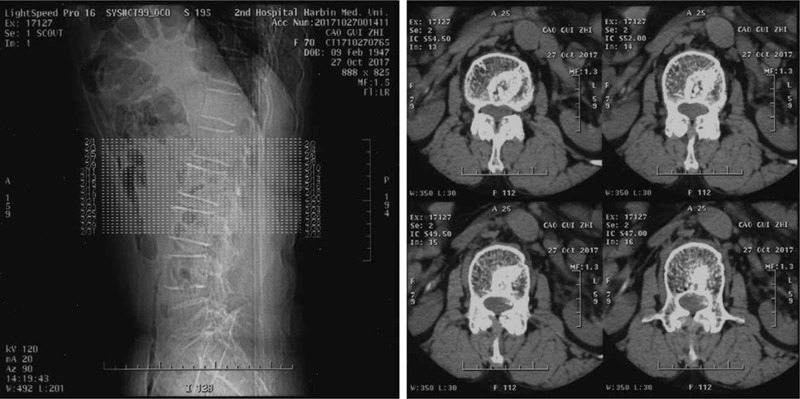
Osteosclerosis led to the formation of a hole in the upper posterior half of the L2 vertebral body. The herniated disc emerged directly from the vertebral body of L2 through a hole in its posterior wall, resulting in the compression of the dural sac.

**Figure 3:**
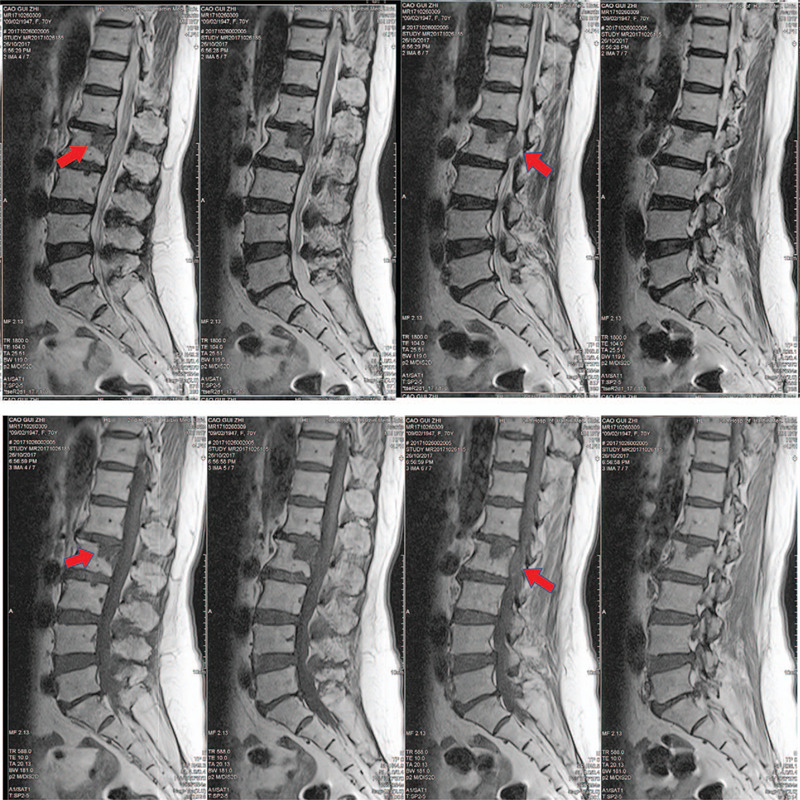
Low signal intensity on the T1WI and T2WI in the upper posterior half of the L2 vertebral body (red arrow). The herniated disc broke through the posterior wall of the L2 vertebral body and was present in the left lateral and dorsal epidural spaces, as shown on the T1WI and T2WI. There was significant lumbar stenosis present at the level of the L2 vertebral bodies secondary to dural sac compression due to the mass.

Radiographs (Fig. 1) showed obvious osteoporosis in the vertebrae, and the endplates of L2, L3, and L4 showed osteosclerosis. Osteosclerosis was also observed in the upper posterior half of the L2 vertebral body. Lumbar computed tomography (Fig. 2) demonstrated that the presence of osteosclerosis had led to the formation of a hole in the upper posterior half of the L2 vertebral body. The herniated disc emerged directly from the vertebral body of L2 through a hole in the posterior wall, resulting in the compression of the dural sac. Magnetic resonance imaging of the lumbar spine showed low signal intensity on T1WI and T2WI in the upper posterior half of the L2 vertebral body. A herniated disc that broke through the posterior wall of the L2 vertebral body was present in the left lateral and dorsal epidural spaces, and showed a low signal intensity on the T1WI and T2WI. Significant lumbar stenosis was present at the level of the L2 and L4/5 vertebral bodies secondary to dural sac compression by the mass (Fig. 3).

**Figure 4:**
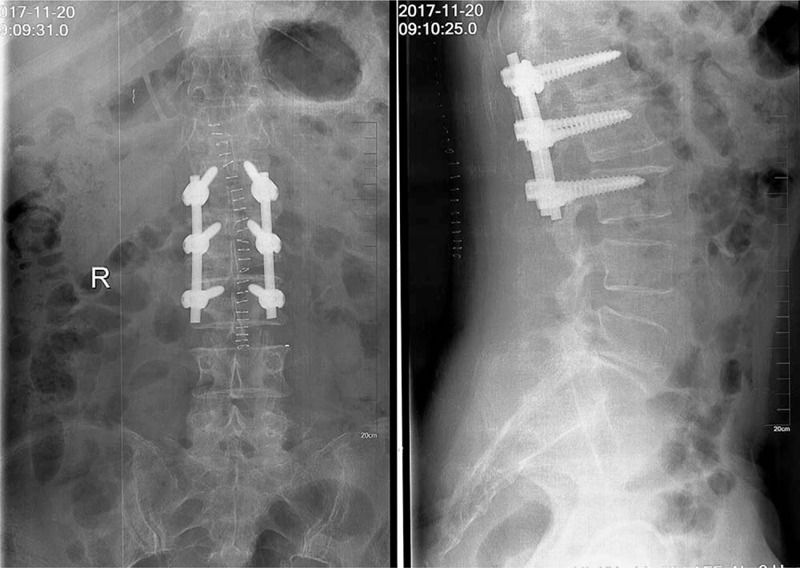
Postoperative radiography and pedicle screw fixation at L1–3.

**Figure 5:**
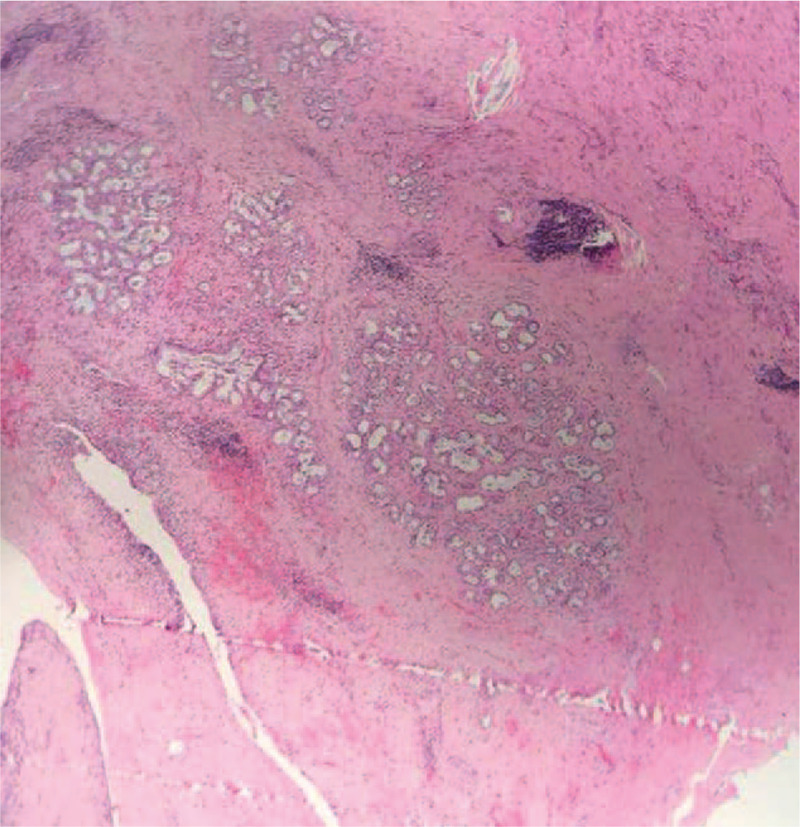
The pathological examination revealed the presence of disc tissue (hematoxylin and eosin stain, × 40).

The patient underwent left hemilaminectomy at the L2 level (Fig. 4). Following medial retraction of the dural sac and the left L2 rootlet, a large crumby mass similar to the nucleus pulposus tissue was found, and the dural sac and L2 left nerve root were seriously compressed. We removed the ruptured disc and bone fragments and searched the epidural space for other compression lesions. Pedicle screw fixation was performed at L1–3 using the Medos International SARL. Lumbar interbody fusion was performed by grafting the bone fragments. The mass was confirmed to be disc tissue by a postoperative pathological examination (Fig. 5).

Postoperatively, the patient's leg pain resolved immediately, and her back pain was eased. The patient recovered normal motor function at 20 days after surgery.

## 3 Discussion

An intervertebral disc is composed of the nucleus pulposus, which primarily consists of type II collagen and accounts for 20% of its overall dry weight, and the annulus fibrosis, which consists of type I collagen fibers and accounts for 70% of its dry weight.^[7,8]^ The pressure within the nucleus pulposus increases when compressive forces are applied across the disc space, leading to a more flattened nucleus that pushes against the circumferentially positioned annular fibers and places them under tension. The integrity of the annulus fibrosis allows it to disperse stresses and constrain the nucleus pulposus within the disc. If the annulus fibrosis is disorganized, the soft nucleus can be pushed through and herniated. The cause of annulus fibrosis destruction may be genetic, trauma, infection, or degeneration associated with aging. Subsequently to annulus fibrosis, the disc may be “protruded”, “extruded”, “sequestrated”, or may even have “migrated”. Alternatively, it may form a Schmorl node.

Schmorl’ nodes were first described in 1927. Preliminary epidemiological studies demonstrated that Schmorl nodes mainly occur in males, and those in the T10-L1 region are associated with higher morbidity than those in the L2-L5 region. However, another study implied that there was no correlation between the number, location, or size of Schmorl nodes and the patient's age or bone density.^[9]^ Schmorl nodes have been defined as a type of herniation of the nucleus pulposus through the cartilage and endplate to the body of the adjacent vertebra.^[10]^ According to the analysis of more than 400 magnetic resonance imaging scans of the lumbar spine performed by Modic in 1988,^[11]^ there are 3 types of endplate changes that can be identified based on T1WIs and T2WIs. Type 1 changes correspond to hypointensity on T1WIs and hyperintensity on T2WIs, representing bone marrow edema and inflammation. Type 2 corresponds to hyperintensity on both T1WIs and T2WIs and represents the conversion of normal red hemopoietic bone marrow into yellow fatty marrow as a result of ischemia. Type 3 changes correspond to hypointensity on both T1WIs and T2WIs, representing a later stage of degeneration characterized by subchondral bone sclerosis.^[11]^ The progression of type 1 to type 2 and type 3 represents the natural history of endplate changes associated with degenerative disc disease. The later stage of endplate degeneration (type 3), when superimposed by external factors (such as trauma) or systemic factors (such as osteomalacia, developmental defects, infection, or Scheuermann disease), can manifest as a complete disruption of the endplate cartilage and extravasation of the disc content into the vertebral body to form Schmorl nodes. However, alterations in the subchondral bone itself (hyperparathyroidism, Paget disease, neoplasms, and osteoporosis) can also result in the formation of Schmorl nodes. In our case, the X-ray results of the elderly patient demonstrated serious osteoporosis, and the lesion developed from the posterior part of the superior endplate of L2, which was surrounded by bone sclerosis. The Schmorl nodes then most likely progressed through the vertebral body to the spinal canal through a hole in the posterior margin, thereby compressing the nerve. We surmised that the reason for this phenomenon was serious osteoporosis. However, another reported case suggested that this defect likely developed in an area of diminished resistance because of the presence of vertebral vascular venous channels.^[5]^ As such the etiological mechanism remains unclear.

**Figure 6:**
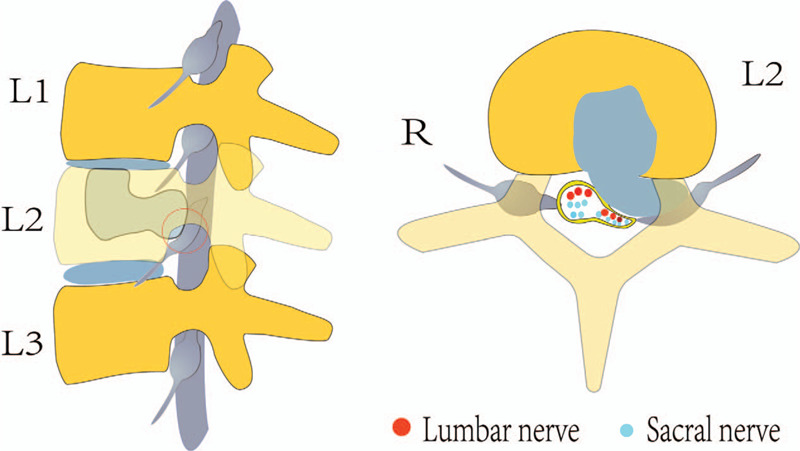
We hypothesized that the herniated disc penetrated through the vertebral body (L2) and not only compressed the L2 nerve root, but also the L4/5 and S1 left nerve root resulting in multiple radiculopathy.

In our case, according to the imaging manifestations, it was clear that the herniated disc penetrated through the vertebral body (L2) and compressed the L2 left nerve root. However, the physical examination suggested that there was also L4/5 and S1 radiculopathy. We hypothesized that in our case, the L4/5 and S1 left nerve roots were also compressed at the L2 lumbar level (Fig. 6), as the L4/5 and S1 nerve root travel down the spinal canal as part of the cauda equine. Within the thecal sac, the more distal sacral nerve roots are situated dorsally, whereas the lumbar nerve roots travel ventrally within the thecal sac prior to exiting the spinal canal via the designated intervertebral foramina. However, the midline septum of the posterior longitudinal ligament is firmly attached to the vertebral body and divides the anterior epidural space into 2 symmetrical portions. Therefore, the disc is strictly lateral unless prominent. The computed tomography results demonstrated that osteosclerosis led to the formation of a hole in the middle upper posterior half of the L2 vertebral body. Therefore, the nucleus pulposus could have herniated through the posterior midline of the body, compressing other nerve roots, such as the L4/5 and S1 nerve roots. The patient's symptoms were relieved after the nucleus pulposus that herniated through the L2 body was removed.

Herein, we report a rare case of Schmorl nodes, which likely progressed through the vertebral body to the spinal canal through a hole in the posterior margin, thereby compressing the nerve. This resulted in pain in the relevant vertebrae as well as distant nerve root radiculopathy. Additional studies are needed for a comprehensive understanding of the pathophysiology underlying this condition.

## Acknowledgments

The authors thank their colleagues for making this case report possible.

## Author contributions

**Conceptualization:** Guangrong Ji, Yongjie Chen.

**Investigation:** Yongjie Chen, Guojun Wei.

**Methodology:** Guangrong Ji.

**Resources:** Guangrong Ji, Zongguang Li, Naichun Yu.

**Supervision:** Guangrong Ji, Zongguang Li, Naichun Yu.

**Writing – editing:** Guangrong Ji, Guojun Wei.

**Writing – original draft:** Yongjie Chen.
